# Hematological Profile in COVID-19 Infection Among Patients in a Tertiary Care Hospital in Tamil Nadu, South India

**DOI:** 10.7759/cureus.30731

**Published:** 2022-10-26

**Authors:** Nayana Chandran, Karthik Sigamani, Altaff Khadeja Bi

**Affiliations:** 1 Pathology, Karpaga Vinayaga Institute of Medical Sciences and Research Centre, Chengalpattu, IND; 2 Clinical Biochemistry, Karpaga Vinayaga Institute of Medical Sciences and Research Centre, Chengalpattu, IND

**Keywords:** c-reactive protein, schistocyte, prognosis, anemia, thrombocytopenia, neutrophil-lymphocyte ratio, lymphopenia, neutrophilia, leukocytosis, leukopenia

## Abstract

Introduction

A wide range of hematological abnormalities has been observed in SARS-CoV-2 infection which is directly related to the disease progression, clinical severity, and mortality among affected individuals. The objective of this study was to evaluate the abnormalities in hematological parameters among severe acute respiratory syndrome coronavirus 2 (SARS-CoV-2) infected patients in a tertiary care hospital in south India.

Methods

This was a cross-sectional study carried out in the pathology department of Karpaga Vinayaga Institute of Medical Sciences and Research Centre, Chengalpattu, Tamil Nadu, India from 1^st^ May 2021 to 30^th^ June 2021. The hematological reports including complete blood count (CBC), neutrophil-lymphocyte ratio (NLR), serum ferritin, serum C-reactive protein (CRP), serum lactate dehydrogenase (LDH), and D-dimer levels of all the blood samples from COVID-19 positive patients were retrieved from the laboratory records. The Leishman-stained peripheral smear findings were also tabulated and analyzed.

Results

Out of 65 patients, 38 (58.5 %) were males and 27 (41.5%) were females with a majority (78.4%) of them being more than 40 years of age. The salient hematological abnormalities were leukopenia (21.5%), elevated NLR (43%), and thrombocytopenia (6.2%). Peripheral smear showed schistocytes (15.4%), neutrophils with ring nuclei (84.6%), and toxic granules (81.5%). A statistically significant association between elevated NLR and serum CRP was seen among male patients. The association between the presence of schistocytes with serum LDH and D-dimer levels was statistically insignificant.

Conclusions

The significant hematological abnormalities in patients with COVID-19 infection were elevated NLR, lymphopenia, thrombocytopenia, and elevated D-dimer levels. Careful evaluation of the hematological parameters will help in categorizing the high-risk cases and thereby initiating early intervention and appropriate intensive care management. This will bring down the morbidity and mortality among COVID-19 patients.

## Introduction

The novel corona viral infection resulting in severe acute respiratory syndrome (SARS) in humans has been declared a global pandemic by the World Health Organisation (WHO) and has affected more than one million people worldwide since its initial outbreak in Wuhan, China in December 2019 [1]. The global pandemic caused by severe acute respiratory syndrome corona virus-2 (SARS-CoV-2) infection in humans has resulted in increased morbidity and mortality worldwide. It continues to be a great challenge to the healthcare system in many countries even today.

Coronavirus disease 2019 (COVID-19) was first isolated from the respiratory airway epithelial cells of humans and was named SARS-CoV-2 [2]. The SARS-CoV-2 is a single-stranded positive-sense ribonucleic acid (RNA) virus that spreads by droplet infection and affects humans by attaching to angiotensin-converting enzyme-2 (ACE-2) receptor in the airway epithelial cells leading to lung parenchymal involvement [3]. While most patients develop an uncomplicated illness, approximately 1% to 4% develop severe lung involvement with a progressive reduction in oxygen saturation requiring admissions to the ICU [4]. The ACE-2 receptors are also distributed in the heart, kidneys, liver, intestine, vascular endothelium, cerebral cortex, and hematopoietic cells. Hence SARS-CoV-2 infection will manifest as a systemic disease with the involvement of multiple organ systems including gastrointestinal, neurological, immunological, cardiovascular, and hematopoietic systems [3].

Several studies have found a wide range of hematopoietic abnormalities in SARS-CoV-2 infection which are directly related to the disease progression, clinical severity, and mortality among the affected individuals [3]. Cytokine storm is characterized by increased levels of cytokines including tumor necrosis factor-alpha (TNF-α), interleukin (IL)-6, etc. in the plasma and is associated with worsening of the COVID-19 patient’s clinical condition. These inflammatory cytokines also affect the hematological system causing abnormalities of complete blood count (CBC) parameters, peripheral smear, coagulation studies, etc. [5].

A wide range of CBC abnormalities in COVID-19 have been observed and they include leukopenia or leukocytosis, thrombocytopenia, lymphopenia, neutrophilia with increased neutrophil-lymphocyte ratio (NLR), eosinopenia and abnormal platelet-lymphocyte ratio (PLR) [5]. Peripheral smear examination revealed abnormal WBC morphology. Neutrophils showed abnormal nuclear segmentation, nuclear projections, fetus-like nuclei (COVID nuclei), and toxic granules. Monocytes showed cytoplasmic vacuoles. Lymphopenia with occasional reactive large granular lymphocytes was seen in some cases. Thrombocytopenia with giant platelets was seen in some patients. The RBCs showed evidence of microangiopathic hemolysis in the form of fragmented RBCs, increased polychromasia, and nucleated RBCs in severe COVID-19 infection [6,7].

However, there is still a paucity of consistent hematological data and the need for extensive research worldwide to establish the various clinically relevant hematological abnormalities that will help in the stratification and management of COVID-19 patients. The objective of the study was to evaluate the abnormalities in hematological parameters among SARS-CoV-2 infected patients in a tertiary care hospital in south India.

## Materials and methods

Setting

This was a cross-sectional study carried out in the pathology department of Karpaga Vinayaga Institute of Medical Sciences and Research Centre, Chengalpattu, Tamilnadu. The study period was two months from 1st May 2021 to 30th June 2021.

Study population

Includes all patients confirmed with COVID-19 infection. A confirmed case of COVID-19 was defined by a positive result on the reverse transcriptase polymerase chain reaction (RT-PCR) assay of a specimen collected on a nasopharyngeal swab.

Inclusion criteria

Blood samples from individuals of all age groups and gender with a positive COVID-19 report by RT-PCR.

Exclusion criteria

Individuals with other causes of fever.

Sample size and sampling

A purposive sampling technique was used for the selection of desired samples according to the inclusion criteria. All the blood samples received from patients with an RT-PCR-confirmed COVID-19 infection in the department of pathology during the two months study period were used for the study.

Protocol

The study was approved by the Institutional Ethics Committee for Human Studies of Karpaga Vinayaga Institute of Medical Sciences and Research Center (approval number: KIMS/F/2021/02). Informed consent was obtained from all patients during blood sample collection. This was a laboratory-based study where the hematological reports including CBC, NLR, serum ferritin, serum C-reactive protein (CRP), serum lactate dehydrogenase (LDH), and D-dimer levels of all the blood samples from COVID-19-positive patients received from 1st May 2021 to 30th June 2021 in the department of pathology were retrieved from the laboratory records. The Leishman-stained peripheral smears of all those patients were reviewed. All the findings were tabulated and analyzed.

Statistical analysis

Data were entered into Microsoft Excel (Microsoft Corp., Redmond, WA, USA) and analyzed using Statistical Package for Social Sciences (SPSS) software version 22.0 (IBM Corp., Armonk, NY, USA). Pearson’s chi-square test was used to evaluate the association between NLR and other serum markers like CRP, ferritin, LDH, and D-dimer levels. A p-value of 0.05 was taken as the cut-off point to determine statistically significant results. Frequency and percentage were calculated for categorical variables like patient demographic data such as age and gender.

## Results

In the present study, a total of 65 patients confirmed with COVID-19 infection were included, out of which 38 (58.5%) were males and 27 (41.5%) were females. The majority of the patients (78.4%) were more than 40 years of age (Table 1).

**Table 1:** Age and gender distribution of patients

Age	Males (n)	Females (n)	Number of patients n (%)
≤ 20	2	0	02 (3.1%)
21 - 30	3	0	03 (4.6%)
31- 40	4	5	09 (13.9%)
41-50	9	5	14 (21.5%)
51- 60	6	8	14 (21.5%)
61- 70	7	7	14 (21.5%)
>71	7	2	09 (13.9%)
Total	38 (58.5%)	27 (41.5%)	65 (100%)

In this study, all the patients including both males and females were categorized into three groups based on hemoglobin levels. Out of the 65 patients, 33 cases (50.8%) had adequate hemoglobin levels of above 12g/dl, around 27 cases (41.5%) had a mild reduction in the hemoglobin value, and only five cases (7.7%) had moderate anemia with hemoglobin levels less than 10g/dl. There were no cases of severe anemia with hemoglobin levels of less than 7g/dl in this study (Table 2).

**Table 2:** Hemoglobin distribution among patients

Hemoglobin (g/dl)	Number of patients	Percentage
<10	05	07.7%
10 - 12	27	41.5%
>12	33	50.8%
Total	65	100%

Patients with a total WBC count of less than 4000/cu.mm were considered as having leukopenia and those with values more than 11,000/cu.mm were considered as having leukocytosis. In this study, 43 cases (66.2%) had a WBC count in the normal range while 14 cases (21.5%) had leukopenia, and eight cases (12.3%) had leukocytosis (Table 3).

**Table 3:** Distribution of total WBC count among patients

Total WBC count (cells/ cu.mm)	Number of patients	Percentage
<4000	14	21.5%
4000 -11000	43	66.2%
>11000	08	12.3%
Total	65	100%

The normal reference range for NLR is 1 to 3. In this study, patients with NLR values of more than 4 were considered to have clinically significant raised NLR. Out of 65 cases, 28 patients (43%) had high NLR values with four cases (6.1%) having markedly raised NLR of more than 10 (Table 4).

**Table 4:** Distribution of NLR among patients NLR: Neutrophil-lymphocyte ratio

NLR	Number of patients	Percentage
< 2	07	10.8%
2 - 4	30	46.2%
5 - 7	16	24.6%
8 - 10	08	12.3%
>10	04	06.1%
Total	65	100%

Patients with a platelet count of fewer than 1.5 lakhs/cu.mm were considered as having thrombocytopenia and those with values more than 4 lakhs/cu.mm were considered as having thrombocytosis. In this study, the majority of cases (92.3%) had platelet count in the normal range while four cases (6.2%) had thrombocytopenia and a single case (1.5%) had thrombocytosis (Table 5).

**Table 5:** Distribution of platelet count among patients

Platelet count (cells/cu.mm)	Number of patients	Percentage
<1.5 lakhs	04	6.2%
1.5 - 4.0 lakhs	60	92.3%
>4.0 lakhs	01	1.5%
Total	65	100%

Peripheral smear examination of the patients revealed several abnormalities in the RBCs, WBC,s and platelets. The RBC abnormalities included normocytic normochromic anemia (9.2%), microcytic hypochromic anemia (7.7%), and the presence of schistocytes (15.4%). The WBCs showed various quantitative abnormalities with neutrophilia being the predominant abnormality seen in 89.2% of cases followed by lymphopenia in 53.8% of cases. The WBCs also showed morphological abnormalities in the form of neutrophils with ring nuclei (84.6%) and toxic granules (81.5%). The platelet abnormalities included thrombocytopenia (6.2%), thrombocytosis (1.5%), and giant platelets (4.6%) (Table 6).

**Table 6:** Distribution of peripheral smear abnormalities among patients

S. No.	Peripheral smear features	Type of abnormalities	Number of cases (%)
1.	RBC abnormalities	Microcytic hypochromic anemia	5 (7.7%)
		Normocytic Normochromic anemia	6 (9.2% )
		Schistocytes	10 (15.4%)
2.	WBC abnormalities	Leukocytosis	8 (12.3%)
		Leukopenia	14 (21.5%)
		Neutrophilia	58 (89.2%)
		Lymphopenia	35 (53.8%)
		Lymphocytosis	1 (1.5%)
		Neutrophils with abnormal nuclear morphology (Ring nuclei)	55 (84.6%)
		Neutrophils with toxic granules	53 (81.5%)
3.	Platelet abnormalities	Thrombocytopenia	4 (6.2%)
		Thrombocytosis	1 (1.5%)
		Giant platelets	3 (4.6%)

The association between NLR and serum biomarkers like LDH, ferritin, D-dimer, and CRP was analyzed among males and females. There was a significant association between increased NLR values and serum CRP levels among male patients in this study. However, among females, there was no significant association between NLR and serum CRP. All other serum biomarkers like LDH, ferritin, and D-dimer were not significantly associated with high NLR values among males (Table 7) and females (Table 8) in this study.

**Table 7:** Association of NLR with serum biomarkers among male patients NLR: Neutrophil-lymphocyte ratio, LDH: Lactate dehydrogenase, CRP: C-reactive protein

		NLR					Total male patients (n=38)	Chi-square test
Bio-marker	Serum value	<2	2 - 4	5 - 7	8 - 10	>10		
LDH	≤248 U/L	2	3	2	0	0	07	P-value 0.138
	>248 U/L	1	15	6	7	2	31	
Ferritin	≤434 ng/ml	2	7	2	1	0	12	P-value 0.378
	>434 ng/ml	1	11	6	6	2	26	
D-dimer	≤500 ng/ml	2	6	2	1	0	11	P-value 0.571
	>500 ng/ml	1	12	6	6	2	27	
CRP	≤5 mg/L	2	1	0	0	0	03	P-value 0.007
	>5 mg/L	1	17	8	7	2	35	

**Table 8:** Association of NLR with serum biomarkers among female patients NLR: Neutrophil-lymphocyte ratio, LDH: Lactate dehydrogenase, CRP: C-reactive protein

		NLR					Total female patients (n=27)	Chi-square test
Bio-marker	Serum value	<2	2 - 4	5 - 7	8 - 10	>10		
LDH	≤247 U/L	3	1	2	0	0	06	P-value 0.069
	>247 U/L	1	11	6	1	2	21	
Ferritin	≤300 ng/ml	4	9	8	0	2	23	P-value 0.057
	>300 ng/ml	0	3	0	1	0	04	
D-dimer	≤500 ng/ml	1	3	3	1	1	09	P-value 0.589
	>500 ng/ml	3	9	5	0	1	18	
CRP	≤5 mg/L	1	2	1	0	0	04	P-value 0.922
	>5 mg/L	3	10	7	1	2	23	

The association of schistocytes in peripheral smear with the serum levels of LDH and D-dimer was assessed among males and females which was statistically insignificant in the present study (Table 9).

**Table 9:** Association of serum LDH and D-dimer levels with the presence of schistocytes among male and female patients LDH: Lactate dehydrogenase

Biomarker	Total male patients (n=38)		Total female patients (n=27)		Chi-square test
	Serum value of biomarkers	Male patients with schistocytes (n=7)	Serum value of biomarkers	Female patients with schistocytes (n=3)	
LDH	≤248 U/L	1	≤247 U/L	0	P-value 0.49
	>248 U/L	6	>247 U/L	3	
D-dimer	≤500 ng/ml	4	≤500 ng/ml	2	P-value 0.778
	>500 ng/ml	3	>500 ng/ml	1	

## Discussion

The magnitude of hematological abnormalities among patients infected with SARS-CoV-2 is of varied nature. It is of utmost importance to be aware of the various hematological abnormalities for proper risk stratification and management of the patients considering the high infectivity of COVID-19 [8]. Majority of studies conducted worldwide reported more male patients compared to female patients similar to the present study where male patients accounted for 58.5% of the cases. Patel et al. [1] from India reported 70% of male patients, Bhuiyan et al. [2] from Bangladesh reported 71% male patients while Araya et al. [8] from Ethiopia reported 62.3% male patients. In this study, 78.4% of patients were more than 40 years of age in concordance with the study by Patel et al. [1]. Bhuiyan et al. [2] reported the median age of patients admitted to ICU as 50 years and the median age of non-ICU patients as 32 years.

Hemoglobin was reduced in 49.2% of cases in this study. Patel et al. [1] from India reported decreased hemoglobin in 36% of cases while Chen et al. [9] from China reported a reduction in hemoglobin in 51% of cases which was almost similar to the present study. A meta-analysis by Lippi et al. [10] revealed low hemoglobin concentrations among COVID-19 patients with severe disease than in mild infections.

Leukopenia was observed in 21.5% of cases while 12.3% of cases showed leukocytosis in the current study. In the study by Patel et al. [1] from India, leukopenia was seen in 4% of cases while leukocytosis was seen in 20% of cases, unlike the present study. However, Wan et al. [11] from China reported leukopenia in 20.7% of patients with COVID-19 infection similar to the present study. 

The NLR defined as neutrophil count divided by lymphocyte count is an indicator of prognosis in patients with pneumonia. Elevated NLR signals the progression of pneumonia in COVID-19 patients and thus serves as an important prognostic parameter among COVID-19 patients [12]. In this study, NLR was elevated in 43% of cases out of which 6.1% cases had marked elevations in NLR of more than 10. Terra et al. [13] from Brazil reported a higher death rate among COVID-19 patients with NLR ≥10. Qin et al. [14] from China reported higher NLR among severe patients compared to non-severe COVID-19 patients. Yan et al. [15] reported elevated NLR as a marker for increased mortality rate among hospitalized patients with COVID-19 infection.

In this study, 6.2% of cases had thrombocytopenia while 1.5% of cases had thrombocytosis. Patel et al. [1] from India reported thrombocytopenia in 16% of cases while thrombocytosis was seen in 8% of cases, unlike the present study. Zhou et al. [16] from China reported thrombocytopenia in 7% of cases almost similar to the present study. There was a higher incidence of thrombocytopenia among the non-survivors than among survivors of COVID-19 infection in the study by Zhou et al. [16]. Various pathogenic mechanisms have been involved in the development of thrombocytopenia that includes a direct effect on platelet production by the cytokine storm associated with SARS-CoV-2 infection, increased consumption of platelets due to microthrombi formation in COVID-19 infection, or due to destruction of platelets by auto-antibodies [17].

The peripheral smear findings in this study were compared to other similar studies carried out in different populations of the world. In a study conducted by Pezeshki et al. [18] from Iran, the most frequent peripheral smear abnormalities were thrombocytopenia (78.7%), smudge cells (67.4%), normocytic normochromic anemia (54%), giant platelets (42.7%), atypical lymphocytes (36%), leukopenia (37.1%), leukocytosis (29.2%) and schistocytes (27%), unlike the present study (Table 10).

**Table 10:** Comparison of peripheral smear abnormalities with the study conducted by Pezeshki et al.

Peripheral smear features	Present study	Pezeshki et al.'s study [18]
RBC abnormalities	Microcytic hypochromic anemia (7.7%)	Microcytic hypochromic anemia (15.7%)
	Normocytic normochromic anemia (9.2%)	Normocytic normochromic anemia (54%)
	Schistocytes (15.4%)	Schistocytes (27%)
WBC abnormalities	Leukocytosis (12.3%)	Leukocytosis (29.2%)
	Leukopenia (21.5%)	Leukopenia (37.1%)
	Neutrophilia (89.2%)	Leukoerythroblastic reaction (9%)
	Lymphopenia (53.8%)	Smudge cells (67.4%)
	Lymphocytosis (1.5%)	Atypical lymphocytes (36%)
	Neutrophils with ring nuclei (84.6%)	Immature neutrophils (14.6%)
	Neutrophils with toxic granules (81.5%)	Large granular lymphocytes (9%)
Platelet abnormalities	Thrombocytopenia (6.2%)	Thrombocytopenia (78.7%)
	Thrombocytosis (1.5%)	Thrombocytosis (2.2%)
	Giant platelets (4.6%)	Giant platelets (42.7%)

In a study of leukocyte morphological changes in COVID-19 by Kannan et al. [7] from India, the neutrophils showed acquired neutrophilic nuclear projections (ANNP), acquired Pelger-Huet anomaly (APHA), toxic granules and myeloid shift to the left while the lymphocytes showed plasmacytoid appearance (plasmacytoid lymphocytes) in comparison to the present study which showed neutrophils with ring nuclei and toxic granules. Another study by Gaffoor et al. [19] from India reported the SARS-CoV-2 induced morphological changes in neutrophils, lymphocytes, and monocytes where the neutrophils showed C-shaped nuclei, pseudo-Pelger-Huet anomaly, toxic granules, and cytoplasmic vacuolations. The morphological abnormalities in the lymphocytes were large granular lymphocytes, plasmacytoid lymphocytes, and atypical lymphocytes while the monocytes showed cytoplasmic granules, vacuolation, and nuclear blebbing [19]. Gaffoor et al. [19] reported a statistically significant association between higher grades of morphological abnormalities in leukocytes and the clinical severity of COVID-19 infection. The cytokine storm associated with SARS-CoV-2 infection is attributed to the development of morphological changes in WBCs.

In a study by Nasab et al. [12], NLR was positively correlated with serum CRP levels in concordance with the present study which showed a significant association between increased NLR values and serum CRP levels among male patients. However, there was no significant correlation between NLR and serum CRP levels among female cases which may be due to the less number of female patients in this study. The combination of NLR and CRP may serve as a valuable early marker to assess the prognosis and evaluate the severity of clinical symptoms among COVID-19 patients [12].

In the present study, the other serum biomarkers in COVID-19 infection which includes serum LDH, ferritin, and D-dimer were not significantly associated with high NLR. Terra et al. [13] reported a higher death rate among COVID-19 patients with NLR ≥10 along with D-dimer levels ≥2µg/ml. Monica et al. [20] reported a statistically significant correlation between elevated NLR and serum ferritin among COVID-19 patients admitted to the ICU.

Schistocytes are fragmented RBCs in the peripheral blood smear of patients suffering from thrombotic microangiopathic diseases. Della Rocca et al. [21] reported a schistocyte count of ≥ 1% in 70% of hospitalized COVID-19 patients and schistocytes may serve as an important biomarker to categorize the high-risk sub-population with latent systemic microvascular damage caused by SARS-CoV-2 irrespective of respiratory symptoms. However, in another study by Pezeshki et al. [18], the presence of schistocytes in peripheral smear had no prognostic significance similar to the present study where there was no statistically significant association between the presence of schistocytes in peripheral smear and the prognostic serum markers like LDH and D-dimer levels.

Limitations

The present study was based on laboratory data. The clinical status of the patients with COVID-19 infection and the radiological features were not included in this study. Further studies incorporating the clinical, radiological, and laboratory data will provide better insight into the relationship between the various hematological parameters and the clinical severity of COVID-19 infection.

## Conclusions

The common hematological abnormalities in patients with COVID-19 infection were elevated NLR, lymphopenia, thrombocytopenia, and elevated D-dimer levels. Some of the hematological parameters including elevated NLR, thrombocytopenia, and lymphopenia have been found to correlate with the clinical severity of the SARS-COV-2 infection in many patients and are useful in monitoring the patients during the course of the disease. Awareness of the hematological abnormalities and their careful evaluation during the disease course will play a vital role in predicting the severity of the clinical illness and thereby guiding early intervention and appropriate intensive care to those who are in need. This will bring down the morbidity and mortality caused by SARS-CoV-2 infection to a considerable extent.
